# The Study of the Inheritance Mechanisms of Myotonic Dystrophy Type 1 (DM1) in Families from the Republic of North Ossetia-Alania

**DOI:** 10.3390/ijms25179734

**Published:** 2024-09-09

**Authors:** Sofya A. Ionova, Aysylu F. Murtazina, Andrey A. Marakhonov, Olga A. Shchagina, Nina V. Ryadninskaya, Inna S. Tebieva, Vitaly V. Kadyshev, Artem O. Borovikov, Evgeny K. Ginter, Sergey I. Kutsev, Rena A. Zinchenko

**Affiliations:** 1 Research Centre for Medical Genetics, Moskvorechie Str. 1, 115522 Moscow, Russia; sofya.aydarovna.g@med-gen.ru (S.A.I.); aysylumurtazina@gmail.com (A.F.M.); marakhonov@generesearch.ru (A.A.M.); schagina@med-gen.ru (O.A.S.); outremal@yandex.ru (N.V.R.); vvh.kad@gmail.com (V.V.K.); borovikov33@gmail.com (A.O.B.); ekginter@mail.ru (E.K.G.); kutsev@mail.ru (S.I.K.); 2 North Ossetian State Medical Academy of the Ministry of Health of the Russian Federation, Pushkinskaya St., 40, Republic of North Ossetia-Alania, 362019 Vladikavkaz, Russia; tebinna@mail.ru; 3 Medical and Genetic Consultation of the Republican Children’s Clinical Hospital of the Republic of North Ossetia-Alania, Barbashova 33A, 362020 Vladikavkaz, Russia

**Keywords:** Republic of North Ossetia-Alania, RNOA, myotonic dystrophy type 1 (DM1), segregation analysis, statistical analysis, population analysis, the *DMPK* gene, anticipation

## Abstract

Myotonic dystrophy type 1 (DM1) is a multisystem disorder with progressive myopathy and myotonia. The clinical study was conducted in the Republic of North Ossetia-Alania (RNOA), and in it 39 individuals from 17 unrelated families were identified with DM1. Clinical presentations varied, including muscle weakness, fatigue, intellectual disability, hypersomnia, ophthalmological abnormalities, and alopecia. Using clinical and genotyping data, we confirmed the diagnosis and enabled the study of CTG-repeat anticipation and DM1 prevalence in the Ossetian and Ingush populations. CTG expansion correlated with age of onset, with clinical severity, and with offspring showing more severe symptoms than parents. In many families, the youngest child had a more severe DM1 phenotype than older siblings. The prevalence was 14.17 per 100,000 in Ossetians and 18.74 per 100,000 in Ingush people, aligning with global data. Segregation analysis showed a higher frequency of maternal transmission. The study highlights the clinical and genetic heterogeneity of DM1 and its dependence on repeat expansion and paternal and maternal age.

## 1. Introduction

Myotonic dystrophy (DM) is a hereditary autosomal dominant neuromuscular disorder which is characterized by progressive muscle weakness and myotonia. There are two types of DM, type 1 (DM1) and type 2 (DM2), in both cases these are repeat expansion diseases. DM1 is the more severe clinical form of the disease [1]. The first mention of DM1 was in 1909 by Hans Steinert, a German neurologist, who first published a series of six cases [2]. DM1 is caused by the expansion of CTG repeat in the 3′-untranslated region (3′-UTR) of the *DMPK* gene. The CTG repeat in 3′-UTR transcribed into GUC repeat in RNA, which is bound by the RNA-binding protein MBNL1, which provides alternative splicing in the fetal-to-adult transition in the heart, muscle, and brain [3]. The expansion of GUC repeat forms a GUC hairpin in RNA, which binds to MBNL1 and sequesters it, which leads to an inability of MBNL1 to participate in the splicing mechanism, resulting in a wide range of proteins being mis-spliced and becoming non-functional [4].

There are many classifications of DM1. Based on the onset of the main symptoms, six subtypes of DM1 may be recognized: pre-mutation, congenital (CDM1), infantile, juvenile, adult, and late-onset DM1 [5,6]. Based on clinical severity, three phenotypes of DM1 are described: congenital, classic, and mild. Congenital DM1 is characterized by severe muscle hypotonia and generalized weakness at birth, intellectual development, and often with respiratory insufficiency and early death [7]. The first DM1 symptoms appear in the prenatal period and are characterized by a significant decrease in the motor activity of the fetus. Classic DM1 is characterized by a combination of symptoms of myopathy (the weakness and atrophy of various muscle groups); myotonia, which occur predominantly in the flexors of the fingers and masticatory muscles; cardiovascular (symptoms of conduction disturbances and ventricular hypertrophy); endocrine-vegetative disorders (hypogonadism, azoospermia, and decreased libido in men and menstrual irregularities, hirsutism, and early menopause in women); and cataracts. Thirty percent of patients have intellectual disability and decreased IQ [8]. A mild form of DM1 is characterized by cataracts and mild myotonia with patients experiencing a normal life span.

DM1 is characterized by genetic heterogeneity that leads to different phenotypic manifestations: a number of repeats from 5 to 38 is normal; a number of repeats from 39 to 50 is considered pre-mutation, in which patients do not present symptoms, but their children are at increased risk of inheriting a larger repeat size and thus having symptoms; from 50 up to about ~100–150 repeats corresponds to a mild form of DM1; more than ~100–150 repeats corresponds to the classical and congenital form of DM1 [9,10]. Moreover, CTG expansion is characterized by somatic instability and epigenetic modifications in different tissues, which complicates performing molecular genetic diagnosis [11,12].

Since 2017, the Laboratory of Genetic Epidemiology at the Research Centre for Medical Genetics (RCMG) has conducted a comprehensive genetic and epidemiological study of the population of the Republic of North Ossetia-Alania (RNOA) [13]. The aim of this study was to investigate the diversity of DM1’s clinical manifestations and the genetic polymorphism of CTG repeats and determine and explore patterns of inheritance of CTG expansions among Ossetian families.

## 2. Results

As part of an epidemiological study, the population of the RNOA was examined. We observed an accumulation of patients with myotonic dystrophy in the Ossetian population. A total of 39 individuals from 17 unrelated families were identified, 35 of them had clinical features of DM1, and 4 relatives of the probands were asymptomatic. For segregation and population analysis, the cohort was supplemented with affected relatives and consisted of 58 patients from 27 nuclear families with DM1 symptoms. Among them, 55 were Ossetians and 3 were Ingush. For population analysis, the observed populations of Ossetians (*n* = 366,748) and Ingush (*n* = 16,008) were applied. We found that the frequency of DM1 among the Ossetian population is 0.00015 (95% CI: 0.00011–0.00020), and the observed prevalence is 1:6666 (15/100,000). The frequency of DM1 among the Ingush population is 0.00019 (considering 95% CI: 0.00004 to 0.00055), and the observed prevalence is 1:5336 (18.74/100,000).

**Figure 1:**
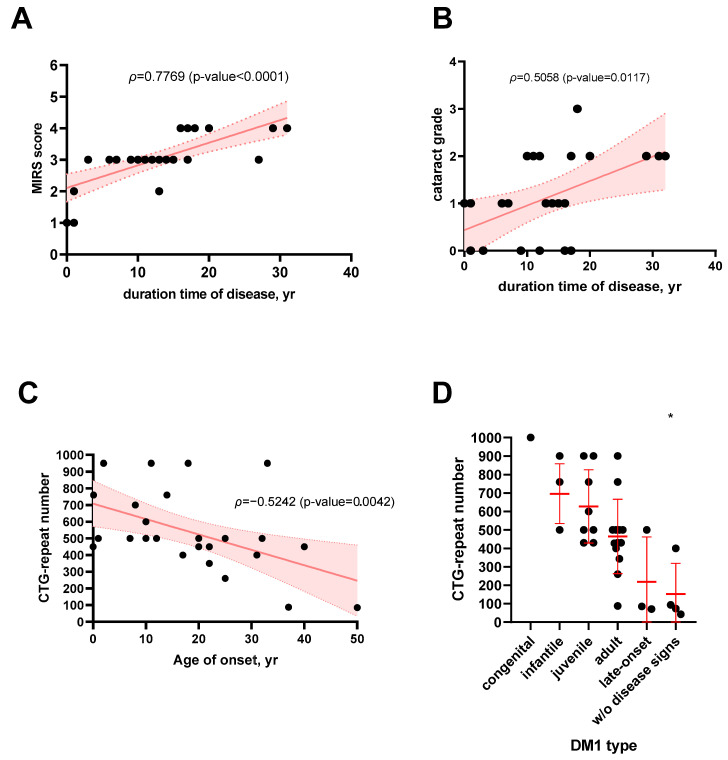
The correlation between duration time of disease in years (yr) and MIRS score (**A**); and cataract grade (0-no cataract, 1-mild, 2-moderate, 3-severe) (**B**); *ρ*—the statistical measure of the strength of a link or relationship between two sets of data; the age of DM1 manifestation and CTG-repeat number (**C**); the plot data of the Kruskal–Wallis H test for comparing different DM1 types with CTG-repeat number (**D**). * marks the type (w/o disease signs), which significantly statistically differs from other DM1 types (infantile and juvenile) (*p* = 0.0198); the median and interquartile range is colored red.

The cohort was formed and consisted of 39 clinically examined people. For all patients, the clinical course of DM1 is different. The patients demonstrated symptoms of DM1: 75% (26/34) of patients had myotonia in different stages—from mild to severe—74% (23/31) presented cataracts, 59% (14/24) demonstrated intellectual disability, 11% (4/36) had hypersomnia, and 41% (13/32) were disabled. Using clinical data, we performed the Spearman rank correlation analysis for 28 patients between the duration of the disease and the severity of clinical signs (cataracts, myotonia) and MIRS score. As a result, we observed a positive correlation between the duration of the disease and severity of cataracts (*ρ* = 0.5920, *p*-value = 0.0014) and an increasing MIRS score (*ρ* = 0.7769, *p*-value < 0.0001) (Figure 1A,B), and we failed to find any correlation between the duration of the disease and myotonia (*ρ* = 0.3229, *p*-value = 0.1005).

The onset age in the cohort ranges from 0 to 50 years. During clinical examination, six previously described phenotypes were present in our cohort: pre-mutation, late-onset, adult, juvenile, infantile, and congenital DM1. Three patients had no information about phenotype (n/d); four patients had no DM1 symptoms (pre-mutation state); six patients had the infantile type of DM1; nine patients had the juvenile type of DM1 and the age of onset for them was early (before 18 years old); three people had the late-onset state; thirteen people had the adult state; and one had the congenital form. There was no information about four patients. The first symptom in most cases was different. The affected parents had a milder clinical course than the affected offspring. They had a late age of manifestation and the different clinical symptoms are moderate cataracts, hand weakness, and general weakness. The offspring demonstrated intellectual and learning disability, developmental delay, muscle pain, muscle weakness and atrophy, moderate and severe myotonia, excessive sweating, upper-limb hyperkinesis, hearing loss, strabismus, optic atrophy, dysphagia, dysarthria, retinal angiopathy, and alopecia. The clinical symptoms are presented in Table S1.

**Figure 2:**
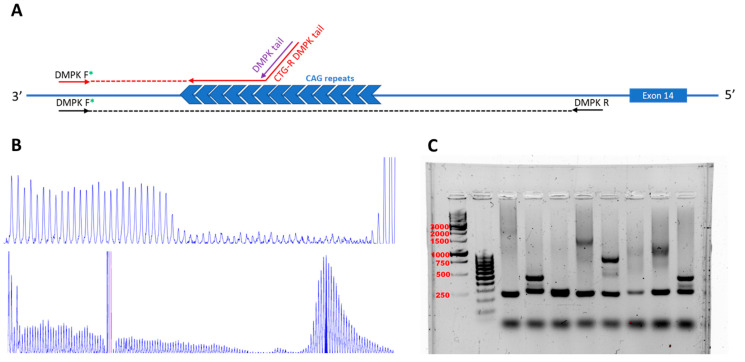
The scheme of primers and localization on 3’UTR of the *DMPK* gene. Primers DMPK F* and DMPK R are used for AGE/PCR, primers DMPK tail and CTG-R DMPK tail together with DMPK F* and DMPK R are used for CE/PCR (**A**). The results of capillary electrophoresis (**B**) and agarose gel (**C**) using the AmplideX^®^ DM1 Dx Kit by which we can identify the number of CTG repeats.

During the study, 37 people from the cohort were genotyped. Determination of the CTG-repeat number was performed using the AmplideX^®^ DM1 Dx Kit (Asuragen, Austin, TX 78,744 USA), which allows detection of the exact number of repeats and is used for DM1 diagnosis (Figure 2). The number of CTG repeats is divided into three subgroups: normal state (5–38 repeats), pre-mutation state (38–50 repeats), and disease manifestation (over 50) [9].

Based on genotyping results, we performed the Spearman rank correlation analysis for 28 patients to determine the link between the age of onset and the number of CTG repeats, and we observed a negative correlation (*p*-value = −0.5242). Patients with the highest number of repeats exhibited DM1 clinical symptoms earlier (Figure 1C). Also, we compared six groups of patients (*n* = 34) with different DM1 phenotypes observed in cohorts with CTG expansion using the non-parametric Kruskal–Wallis H test and DM1 phenotype groups with each other using Dunn’s multiple comparisons test. The results demonstrated that phenotype groups depend on the number of CTG repeats with an approximate *p*-value = 0.0024. Dunn’s multiple comparisons test demonstrated that the pre-mutation phenotype (w/o disease signs) significantly differs from the infantile phenotype (*p*-value = 0.0177) and from the juvenile phenotype (*p*-value = 0.0490) (Figure 1D).

**Figure 3:**
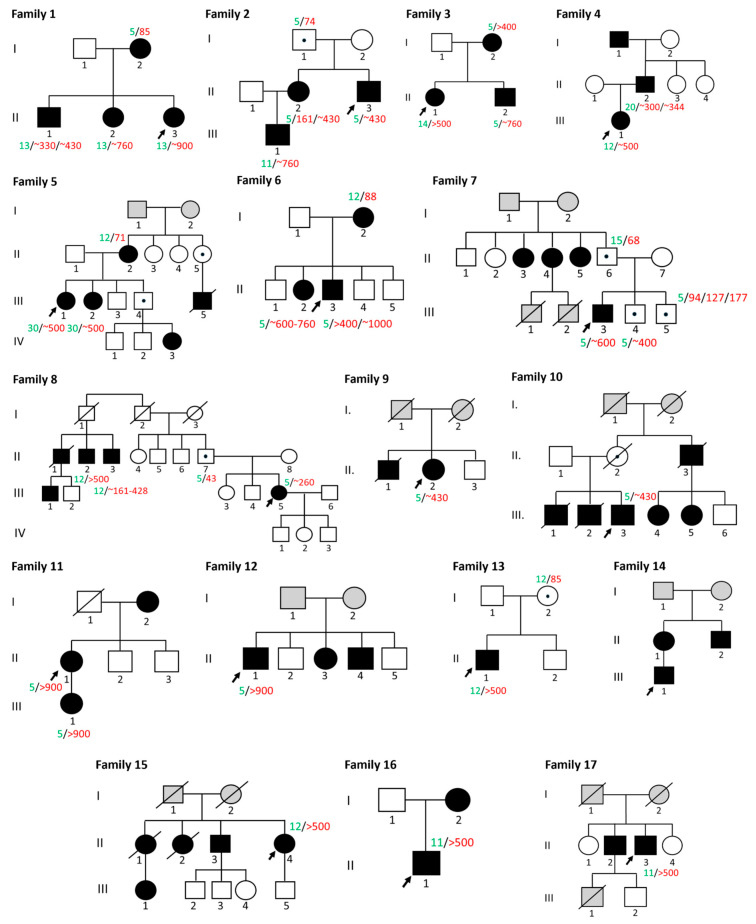
The pedigree of all 17 families with genotyping results. Affected family members are filled in black, family members without information about DM1 (affected or not affected) are filled grey color. Normal number of repeats marked in green (5–35), pre-mutation and CTG expansion marked in red (>35).

Based on the clinical and genotyping results and pedigree description (Figure 3), several patterns were identified. First, the CTG expansion correlated with the severity of the clinical picture and with an increase in generation in a family. In 11 families (1, 2, 3, 4, 5, 6, 7, 8, 10, 13, and 16), the offspring demonstrated CTG expansion and clinical symptoms were more severe compared to their parents. In families 2, 7, and 10, parents in the 1st generation were without DM1 symptoms, whereas in families 2, 7, and 8 the father was the carrier of DM1; in family 10, the mother was the carrier. In families 3, 4, 5, 6, and 16, the parents demonstrated milder DM1 symptoms compared to the offspring (in families 3, 5, 6, and 16 the mother is affected, and in family 4, the father is affected). In families 1 and 13, the female parents demonstrated only cataracts which may not be associated with DM1. In families 5, 9, 10, and 15, the children died due to DM1; the age at death is unknown (Figure 3).

Second, we observed that the youngest child in the family had a more severe DM1 phenotype than older affected siblings and clinical severity is correlated with CTG expansion. This tendency is clearly detected in families with two or more children—families No. 1, 2, 3, and 6. Nevertheless, in some families, this tendency is not observed (families No. 5, 7, 9, 10, and 15). In family 5, in the second and third generations, the younger children demonstrated a milder clinical picture of DM-1; the oldest proband (III-1) during the examination was observed to have ptosis, cataracts, and weakness of the proximal muscles, diffuse hypotrophy of the upper and lower extremities, muscle hypotonia; the age of onset was 20. The younger affected sibling (III-2) also displayed weakness of the proximal muscles and facial muscles, ptosis, but myotonia was less pronounced; the age of onset was 32. The youngest male sibling (III-4) had no DM1 symptoms. In family 7, proband III-3 was the oldest child in the nuclear family, he had symptoms of DM1, and the largest expansion of CTG repeats compared to his siblings, who did not demonstrate DM1 symptoms. In families 9, 10, and 15, older children were affected and died because of a congenital form of DM1, and the youngest children had a mild form of DM1 or did not have DM1 symptoms.

It is known that DM1 is an autosomal dominant disease, the segregation frequency of which is 0.5. We performed a segregation analysis that allowed us to compare observed and expected segregation frequencies of DM1 in 27 nuclear families, which consisted of 79 healthy people and 58 people with DM1. The segregation frequency was 64.56%. We analyzed the inheritance of a pathogenic allele from mother and father. As a result of the analysis, we showed that the segregation frequency in families in which the pathogenic allele was transmitted from the father was 0.56, which corresponds to the expected value for a dominant type of inheritance of 0.5 (χ^2^ = 0.24). In the case of transmission of the pathogenic allele from the mother, the segregation frequency in these families was 0.70, which exceeds the expected 0.5 (χ^2^ = 4.13).

## 3. Discussion

DM1 is a multisystem disorder that affects skeletal and smooth muscle and is characterized by progressive myopathy and myotonia. During the study in RNOA for several years, doctors from RCMG clinically observed the Ossetian population (366,748 Ossetians and 16,008 Ingushes) and identified among them 39 individuals from 17 unrelated families with DM1, of which 4 were asymptomatic at the time of clinical examination. Initially, pediatric patients visited doctors with a diagnosis of mental retardation, but during clinical examination the diagnosis was changed to DM1. The clinical picture is variable. The patients in our cohort presented muscle weakness, fatigue, intellectual disability, hypersomnia, ophthalmological abnormalities (cataract, strabismus, ptosis), hair loss, and respiratory insufficiency. We used phenotype and genotype classifications that help to describe the clinical picture, diagnose DM1, and establish clear correlations between repeat size and disease phenotype. We used statistical analyses to identify the relationship between the number of CTG repeats and clinical DM1 symptoms. Using Spearman’s rank correlation method, we demonstrated that clinical symptoms worsen with the duration of DM1—cataract grade and MIRS score—which is reflected in clinically recognized distal to proximal progression of the muscular involvement in DM1. Also, gastrointestinal and cardiac complications are common in patients with DM1 [14,15,16]. The prevalence and severity of these complications can vary, highlighting the importance of diagnosis for better management of DM1. Unfortunately, the patients in the cohort did not complain of cardiac and gastrointestinal involvement, so we did not have the opportunity for them to be examined them by these doctors as part of the investigation. Patients underwent annual medical examinations, and no cardiac and gastrointestinal pathologies were described there.

Thirty seven of 39 patients were genotyped due to the availability of biomaterial and voluntary informed consent. For genotyping AmplideX^®^ DM1 Dx Kit was used, which allowed us to determine the most accurate CTG number and confirm the diagnosis. DM1 is an expansion disease, where CTG repeats increase in each generation, and the increasing number of repeats is positively correlated with DM1 severity and with the age of onset [17,18]. We observed CTG expansion in each generation in 11 families from 17; for the other families, we had insufficient data (9, 11, 12, 14, 15, and 17). Based on Spearman’s rank correlation analysis and the Kruskal–Wallis H test, we demonstrated that the age of onset is early, and the phenotype is severe in patients with CTG expansion. Using Dunn’s multiple comparisons test for non-parametric comparison we demonstrated that the pre-mutation group of patients without clinical signs is statistically significant and differs with infantile and juvenile phenotypes (*p*-value < 0.05). However, other phenotypes are not significantly different (*p*-value > 0.05) which may be due to the small phenotype groups.

The overall prevalence of DM1 was estimated as 12.25 cases per 100,000 (95% CI: 7.50–18.06) [19] and the average prevalence can range between 5 and 20 per 100,000 individuals, and the estimated prevalence may be higher in the founder population [20]. The prevalence of DM1 is 1:6666 (or 15 per 100,000) for Ossetians and 1:5336 for Ingush people (or 18, 74 per 100,000), which correlates with the literature data.

The segregation analysis was performed for 27 nuclear families. Segregation analysis by phenotype is the method of correspondence between the distribution of affected and healthy family members and the expected type of inheritance of the disease [21]. It is known that DM1 is an autosomal dominant inheritance disease, and the frequency of pathogenic allele transmission would be 0.5. We demonstrated that the frequency in the Ossetian population is 64.56%, which is not statistically significantly different from 50%.

Previously, it was considered that DM1 is only maternally transmitted [22]. However, in the last few years, it has been demonstrated that DM1 is also paternally transmitted [23,24]. We decided to perform the segregation analysis to explore from whom the pathogenic allele is transmitted. For segregation analysis, we divided families into two groups—the pathogenic allele is inherited from the mother (*n* = 13) and the father (*n* = 9). The segregation frequency for maternal transmission of pathogenic alleles does not correspond with the frequency of the dominant type of inheritance (0.70) in contrast with paternal inheritance (0.56). So, we demonstrated that women transmit the pathogenic allele more often than men. This is probably due to several reasons. Firstly, there are more women than men in RNOA (46% for men and 54% for women, the ratio is 0.92:1.08). Moreover, the proportion of women of fertile age is 2.69% higher than that of men (161,950 people or 50.68% of the total number of people from RNOA are aged 15 to 49 years). Secondly, the difference in puberty between men and women. Females are born with first-order oocytes, which remain in this state in the ovary until the onset of puberty. After puberty, first-order oocytes enter meiosis and begin to divide. Probably, the repeat expansion occurs in the quiescent M1 phase of the primary oocyte during meiosis and, as the female ages, this process may become worse [7,22,25]. So, the CTG expansion during meiosis of the primary oocyte in women is more likely to lead to DM1 than during spermatogenesis, which occurs constantly throughout life. This also explains that the length of the CTG expansion, and the severity of the disease, correlates with the maternal age. We observed this tendency in four families with two or more children in which the youngest child is more severe compared to their older siblings. Thirdly, affected males with the highest CTG-repeat expansions are infertile and are likely unable to inherit the pathogenic allele from their offspring [26], as opposed to females with DM1 who may be fertile but are at a high risk of pregnancy complications [27]. Fourthly, the difference in segregation analysis may be due to epigenetic factors. It is known that DMPK locus methylation is confirmed by CTCF factors. The methylation of upstream CTG repeats is almost always maternally derived from childhood onset, and these epigenetic changes may contribute to the development of more severe forms of the disease [28]. This also explains that the predominantly congenital form is maternally transmitted. However, there are a few publications that demonstrate paternal transmission in the congenital form [29,30].

Due to the investigation in RNOA, we were able to analyze 17 Ossetian families with DM1. We determined the prevalence of DM1 in the Ossetian population, which is similar to the European population. We demonstrated that DM1 is characterized by clinical and genetic heterogeneity. Firstly, clinical and genetic polymorphism is explained by variable phenotypes—from mild to congenital, and differential diagnosis is necessary. Secondly, heterogeneity is explained by CTG expansion with each generation, which correlates with clinical severity of DM1. Thirdly, heterogeneity is explained by the complexity of inheritance mechanisms—the pathogenic allele is inherited from the father with 0.5 frequency. However, the inheritance of pathogenic alleles from the mother is more frequent (0.70). We demonstrated that disease severity depends on mother’s age.

Based on clinical and genotyping results, we were able to confirm DM1, investigate the mechanisms of CTG anticipation in the Ossetian families, the correlation between CTG expansion and the clinical picture and age of onset, the frequency of DM1 in Ossetian and Ingush populations, and inheritance of a pathogenic allele from all families by segregation analysis.

## 4. Materials and Methods

**Patients and methods:** The study included 39 individuals from 17 families. All of them, except one Ingush family, were of Ossetian origin. The gender ratio was 1.07:0.93 (21 men and 18 women), the median of the age data was 34, and the internal scatter of the age data was 77. The patients underwent clinical examination during expeditionary research by a neurologist, ophthalmologist, pediatrician, and geneticist. Comprehensive clinical evaluation including assessment using Muscular Impairment Rating Scale (MIRS) was performed for 33 patients. The study was reviewed and conducted according to the Declaration of Helsinki and approved by the Institutional Review Board of the Research Centre for Medical Genetics, Moskvorechie str., 1, Moscow, Russia (protocol No. 2017-4/1, dated 4 May 2017). Genetic testing was performed for 37 patients. Segregation analysis was performed on nuclear families, which numbered 27 and included 58 probands and their affected siblings. Statistical analysis was performed for 28 patients.

**DNA extraction:** 37 out of 39 participants were available for genetic testing. They signed the informed written consent form (or responsible consent form for infant probands) as anonymous participants of the study and donors of biological materials. Peripheral blood samples were collected, and genomic DNA was isolated using a HiPure Blood DNA Kit (Magen Company, 510,530 Guangdong, China) according to the manufacturer’s recommendations. Genetic testing was performed in the Laboratory of Genetic Epidemiology of RCMG (Moscow, Russia).

**Genetic testing:** For identification of the number of CTG repeats in 3′-UTR of the *DMPK* gene, we used the AmplideX^®^ DM1 Dx Kit (Asuragen, Austin, TX 78744, USA), based on PCR followed by the agarose gel electrophoresis (AGE) and capillary electrophoresis (CE) methods (PCR/AGE and PCR/CE) according to the manufacturer’s recommendations. For CTG repeats below 200, it is recommended to use CE after PCR, which allows us to determine the accurate number of CTG repeats. The kit provides recommendations for deciphering bands after AGE and recommends the use a correction factor for the conversion of size in the base pairs to the number of CTG repeats for each allele. The correction factor was calculated using the formula presented in the manual for the kit. In the kit, it is recommended to use the calibration equation to derive the size and mobility correction factors specific to each set of CE parameters. For CTG repeats of more than 200, it is recommended to use AGE after PCR. Interpretation of gel images may not be accurate due to mosaicism or polymerase errors during PCR, and we observed this as smears in the gel images. The smear is assumed to be comprised of multiple unresolved fragments derived from high levels of repeat instability.

**Population study:** The population study was performed on healthy 366,748 Ossetians and 16,008 Ingush, which were examined during the study. The prevalence of DM1 among the Ossetian and Ingush populations in RNOA was estimated using WINPEPI software (version 11.65) [31]. The confidence interval (CI) for carrier frequency was calculated using Fisher’s method.

**Segregation analysis:** Segregation analysis was performed for 27 nuclear families to determine the frequency of affected people among the population. Segregation analysis also was performed to compare the expected and observed frequency of DM1 among the Ossetian population. For them, we used a chi-squared test for hypothesis testing. Information for compiling pedigrees was collected from probands and their relatives during the examination and recorded according to a generally accepted scheme.

**Statistical analysis:** The correlation analysis was performed for 28 people from the cohort (*n* = 37) to study relationships between the duration of the disease and clinical signs (cataracts, myopia) and MIRS score. The duration time was calculated by subtracting the age of onset from the age of examination. Clinical signs were divided into grades according to severity (1—mild; 2—moderate; 3—severe). People without information about clinical signs (n/d) were not included in the analysis. The correlation analysis was calculated using the Spearman’s rank correlation method.

The Kruskal–Wallis H test was used to compare two independent groups: DM1 phenotype and CTG expansion for 34 patients. For the other 3 patients, the phenotype or CTG number of repeats were unknown (n/d). The patients were divided into six DM1 phenotype groups (congenital—1 patient, infantile—6, juvenile—8, adult—14, late-onset—3, w/o disease signs—4) with different numbers of CTG repeats. We used Dunn’s multiple comparisons test to compare phenotypes with each other. All graphs were constructed using the GraphPad Prism 8.0 tool.

## 5. Conclusions

The study of the Ossetian and Ingush populations revealed a DM1 prevalence comparable to global figures, with significant clinical and genetic heterogeneity. Using genotype and clinical data, we were able to confirm the diagnosis and elicit a statistical analysis between CTG-repeat expansion and disease severity, with a higher frequency of maternal transmission observed. These findings underscore the importance of considering genetic and phenotypic variability in the diagnosis and management of DM1. This work was performed for the first time and is of great importance for the further diagnosis of DM1 among Ossetians and other populations.

## Data Availability

The datasets used and/or analyzed during the current study are available from the corresponding author on reasonable request.

## Supplementary Materials

The supporting information can be downloaded at https://www.mdpi.com/article/10.3390/ijms25179734/s1.
