# Supplementary material for: The Study of the Inheritance Mechanisms of Myotonic Dystrophy Type 1 (DM1) in Families from the Republic of North Ossetia-Alania
Source: Int J Mol Sci. 2024 Sep 9;25(17):9734. doi: 10.3390/ijms25179734 (PMC11395446; doi:10.3390/ijms25179734)
Supplement: Supplementary file 1 [file ijms-25-09734-s001.zip › Table S1.pdf]

Table S1. Clinical and genetic data of 39 patients with DM1.

№ family	Patient	Gender	Nationality	Family member	1 allele	2 allele	Mosaicism	Age of examination (years)	Age of onset (years)	Phenotype	First symptom	Main complaint	Disabled	Intellectual disability	MIRS score	Myotonia	Cataract	Additional features
**1**	I.1	f	Osset.	Mother	5	85		51	50	late-onset	cataract	cataract	no	no	2	mild	mild	
	II.1	m	Osset.	Sib	13	~330, ~430	medium	29	22	adult	alopecia	hypersomnia	no	no	3	moderate	mild	excessive sweating
	II.2	f	Osset.	Sib	13	~760		26	14	juvenile	frequent stumbles	hypersomnia, fatigue	yes	yes	3	severe	no	
	II.3	f	Osset.	Proband	13	~900		12	2	infantile	intellectual disability	intellectual disability	yes	yes	3	severe	moderate	upper limb hyperkinesis
**2**	I.1	m	Osset.	Father	5	74		67	n/a	pre-mutation	no	no	no	no	1	no	intraocular lens	diabetic retinopathy
	II.2	m	Osset.	Sib	5	161, ~430	low	47	20	infantile	feet deformity, motor development delay	intellectual disability	yes	n/d	4	mild	no	
	II.3	m	Osset.	Proband	5	~430	low	33	20	adult	hand muscle stiffness	hand muscle stiffness	no	no	2	severe	mild	early hearing loss
	III.1	f	Osset.	Nephew	11	~760		16	1 mo	adult	hand weakness	hand weakness, muscle stiffness	yes	no	3	severe	intraocular lens	
**3**	I.2	f	Osset.	Mother	5	>400	medium	35	22	adult	hand muscle stiffness	hypersomnia, fatigue	yes	no	3	severe	n/d	hearing loss
	II.1	f	Osset.	Proband	14	>500	high	15	1	infantile	gait disturbance	hypersomnia	yes	yes	3	mild	mild	excessive sweating
	II.2	m	Osset.	Sib	5	~760		9	1 mo	infantile	feet deformity	intellectual disability	yes	yes	3	no	no	strabismus, optic atrophy, hearing loss

Table S1. Continue.

№ family	Patient	Gender	Nationality	Family member	1 allele	2 allele	Mosaicism	Age of examination (years)	Age of onset (years)	Phenotype	First symptom	Main complaint	Disabled	Intellectual disability	MIRS score	Myotonia	Cataract	Additional features
**4**	II.2	m	Osset.	Father	20	~300, ~344	low	42	n/d	adult	hand weakness	hand weakness	no		3	moderate	n/d	
	III.1	f	Osset.	Proband	12	~500		16	7	infantile	hand muscle stiffness	hand weakness, cognitive disorder	no		3	severe	no	
**5**	II.2	f	Osset.	Mother	12	71		82	n/d	late-onset	n/d	general weakness	no		4	moderate	n/d	
	III.1	f	Osset.	Proband	30	~500	high	51	20	adult	ptosis	proximal weakness	no		4	severe	moderate	
	III.2	f	Osset.	Sib	30	~500		49	32	adult	n/d	proximal weakness	yes		4	mild	moderate	
**6**	I.2	f	Ingush	Mother	12	88		37	37	adult	fatigue, hair loss	fatigue, hair loss	no	no	1	no	mild	
	II.2	f	Ingush	Sib	5	~600-760	high	14	8	infantile	learning disabilities	learning disabilities	no	yes	3	mild	mild	
	II.3	m	Ingush	Proband	5	>400, ~1000	high	11	0	congenital	respiratory insufficiency	steppage, lower leg muscle atrophy, learning disabilities	yes	yes	3	mild	moderate	asymmetry
**7**	II.6	m	Osset.	Father	15	68		n/d	n/d	n/d	n/d	n/d	n/d	n/d	n/d	n/d	n/d	
	III.3	m	Osset.	Proband	5	~600		13	10	juvenile	muscle weakness	muscle weakness	no	n/d	3	moderate	no	
	III.4	m	Osset.	Sib	5	~400		10	n/a	pre-mutation	no	no	no	n/d	1	no	no	
	III.5	m	Osset.	Sib	5	94, 127, 177	low	5	n/a	pre-mutation	no	no	no	n/d	1	no	no	

Table S1. Continue.

№ family	Patient	Gender	Nationality	Family member	1 allele	2 allele	Mosaicism	Age of examination (years)	Age of onset (years)	Phenotype	First symptom	Main complaint	Disabled	Intellectual disability	MIRS score	Myotonia	Cataract	Additional features
**8**	II.2	m	Osset.	Cousin	12	>500	high	49	49	adult	n/d	general weakness	yes	no	4	moderate	moderate	optic atrophy
	II.3	m	Osset.	Cousin	12	~161-428	high	51	51	adult	hand weakness	general weakness	yes	no	4	moderate	moderate	optic atrophy
	II.7	m	Osset.	Father	5	43		73	73	pre-mutation	no	no	no	no	1	no	moderate	
	III.5	f	Osset.	Proband	5	~260	low	40	40	adult	hand muscle stiffness	hand muscle stiffness, dysphagia	no		3	severe	mild	dysphagia
	III.1	m	Osset.	Cousin	n/d	n/d		28	15	juvenile	n/d	n/d	n/d	n/d	n/d	n/d	n/d	
**9**	II.2	f	Osset.	Proband	5	~430	low	49	17	juvenile	n/d	n/d	n/d		n/d	n/d	n/d	
**10**	III.3	m	Osset.	Proband	5	~430	low	58	40	juvenile	n/d	n/d	n/d	n/d	n/d	moderate	moderate	strabismus
**11**	II.1	f	Osset.	Proband	5	>900	high	42	33	adult	muscle pain	lower leg weakness,	no	n/d	3	n/d	n/d	
	III.1	f	Osset.	Daughter	5	>900		12	11	juvenile	muscle pain	muscle pain	no	n/d	1	no	no	
**12**	II.1	m	Osset.	Proband	5	>900		30	18	juvenile	n/d	hand weakness	no	n/d	3	severe	moderate	juvenile
**13**	I.2	f	Osset.	Mother	12	85		68	n/d	n/d	n/d	n/d	n/d	n/d	n/d	n/d	moderate	n/d
	II.1	m	Osset.	Proband	12	>500	high	28	12	juvenile	fatigue	general weakness	yes	n/d	4	severe	mild	alopecia
**14**	III.1	m	Osset.	Proband	n/d	n/d	n/d	32	n/d	n/d	no	no	no	n/d	3	severe	moderate	
**15**	II.4	f	Osset.	Proband	12	>500	high	54	25	adult	cataract	general weakness	yes	n/d	4	moderate	intraocular lens	
**16**	II.1	m	Osset.	Proband	11	>500	high	27	10	juvenile	hand weakness	no	n/d	3	no	no	dysarthria, retinal angiopathy	juvenile
**17**	II.3	m	Osset.	Proband	11	>500	high	55	n/d	late-onset	n/d	n/d	n/d	n/d	n/d	mild?	n/d	late-onset
